# A probabilistic model of human variability in physiology for future application to dose reconstruction and QIVIVE

**DOI:** 10.3389/fphar.2015.00213

**Published:** 2015-10-12

**Authors:** Kevin McNally, George D. Loizou

**Affiliations:** Health and Safety LaboratoryBuxton, UK

**Keywords:** human variability, physiology, PBPK, Bayesian, QIVIVE

## Abstract

The risk assessment of environmental chemicals and drugs is undergoing a paradigm shift in approach which seeks the full replacement of animal testing with high throughput, mechanistic, *in vitro* systems. This new approach will be reliant on the measurement *in vitro*, of concentration-dependent responses where prolonged excessive perturbations of specific biochemical pathways are likely to lead to adverse health effects in an intact organism. Such an approach requires a framework, into which disparate data generated by *in vitro, in silico*, and *in chemico* systems can be integrated and utilized for quantitative *in vitro*-to-*in vivo* extrapolation (QIVIVE), ultimately to the human population level. Physiologically based pharmacokinetic (PBPK) models are ideally suited to this and are needed to translate *in vitro* concentration- response relationships to an exposure or dose, route and duration regime in human populations. Thus, a realistic description of the variation in the physiology of the human population being modeled is critical. Whilst various studies in the past decade have made progress in describing human variability, the algorithms are typically coded in computer programs and as such are unsuitable for reverse dosimetry. In this report we overcome this limitation by developing a hierarchical statistical model using standard probability distributions for the specification of a virtual US and UK human population. The work draws on information from both population databases and cadaver studies.

## Introduction

The approach to assessing the risk to human health posed by exposure to chemical hazards has been largely unchanged in the past 50 years (Bhattacharya et al., 2011). The established protocol is based upon observation of an adverse response in a homogeneous animal population subjected to a high dose. An extrapolation to an acceptable dose for human exposures is then made by applying a number of conservative assessment factors. A radical departure from the established protocol was proposed by the US National Research Council in the ground breaking document “Toxicity Testing in the twenty-first Century: A Vision and a Strategy” (National Research Council, 2007). This approach defines intracellular “toxicity” pathways, which are innate sub-cellular biochemical pathways that may be disturbed by environmental stressors. Successful application of this new vision will be reliant on the measurement *in vitro*, of concentration-dependent responses where prolonged excessive perturbations of toxicity pathways are likely to lead to adverse health effects in an intact organism (National Research Council, 2007; Bhattacharya et al., 2011; Krewski et al., 2011; Basketter et al., 2012). Computational systems biology pathway (CSBP) models provide the quantitative description of how multiple cellular response (toxicity) pathways are perturbed following exposure to environmental stressors. However, a framework into which disparate data generated using *in vitro, in silico*, and *in chemico* systems, can be integrated and utilized for quantitative *in vitro*-to-*in vivo* extrapolation (QIVIVE) is required. Physiologically based pharmacokinetic (PBPK) models are ideally suited for this and are necessary to link CSBP models with external exposure or dose (National Research Council, 2007; Blaauboer, 2010; Bhattacharya et al., 2011; Krewski et al., 2011; Basketter et al., 2012). PBPK models are independent, structural models, comprising compartments that correspond directly and realistically to the organs and tissues of the body connected by the cardiovascular system (Rowland et al., 2004; National Research Council, 2007; Zhao et al., 2011).

PBPK models may contain many parameters, particularly so if it is necessary to describe multiple routes of exposure, and the mathematical description of the underlying biology is complex; for example, at least three additional parameters are required for each discretely defined compartment. The addition of a CSPB model to relate a perturbed toxicity pathway observed at the cellular level to an exposure or dose regime requires a significant additional tier of complexity and additional parameters. This mathematical description for the “mean individual” is a significant challenge in itself and at the time of writing the process of linking the endpoint in a perturbed pathway described by a CSPB model to an external dose has yet to be successfully demonstrated. To be of practical use, the methodology needs to be applied at the population level and therefore a probabilistic description of human variability is an essential component.

**Figure 1:**
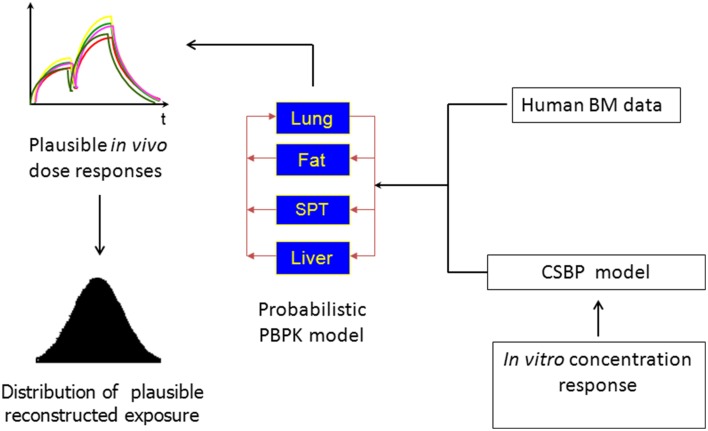
**The reconstruction of exposure from *in vitro* concentration response or biological monitoring data**.

A similar class of problems, referred to in the literature as reverse dosimetry, whereby an external dose is estimated using biological monitoring data from breath, hair, blood or urine biomarker data has been addressed over the last decade (Sohn et al., 2004; Tan et al., 2006a,b; Allen et al., 2007; Lyons et al., 2008; Mosquin et al., 2009; McNally et al., 2012). In this class of problems population-based estimates of exposure that account for human inter-individual variability, both in the modeling of chemical disposition and in the description of plausible exposure conditions can be achieved using Bayesian inference in conjunction with PBPK modeling. McNally et al. (2014) noted that the processes of reverse dosimetry using biological monitoring data and QIVIVE share some important similarities. Specifically, both belong to a class of inverse problems based around a PBPK model, and for inference at a population level a probabilistic description of human variability is an essential component (Figure 1). Difficulties encountered in reverse dosimetry applications will inevitably be exacerbated in a more complex QIVIVE application.

A common feature of reverse dosimetry problems is that, after accounting for all known sources of uncertainty/variability, the range of external exposures that is consistent with measurements is typically wide. Biologically plausible limits on the parameters of the PBPK model may be encoded via informative prior distributions. However, within these limits a potentially wide range of parameter sets (and hence unknown external doses) may offer a similar quality of fit to the available data. Bayesian inference still allows the problem to be resolved and knowledge (or lack of knowledge) about model parameters including ranges, central values and measures of dispersion is described probabilistically. QIVIVE models that require a mathematical description of sub-cellular activity will exacerbate the problem^1^. Given that a wide range of parameter sets result in similar dose-response curves for comparison with measurements, more intensive sampling of this curve offers only very limited scope for discrimination between parameter sets. However, there is a scope for substantial improvements in the mathematical description of human variability and hence discrimination between parameter sets is achieved through the prior distribution.

The present approach to describing variability in human physiology is fairly crude in the context of reverse dosimetry. The problem of describing variability in the human population is converted to a problem of describing variability, via probability distributions, in individual organs and tissues (e.g., adipose, muscle, liver, brain etc.) or aggregated compartments (rapidly and slowly perfused tissues). Masses and blood flows are discretely defined using representative biological data. The specification of masses and flows can be direct with probability distributions assigned to masses and flows of individual organs or aggregated compartments (Sohn et al., 2004) or more typically as a proportion of body weight and cardiac output, respectively (Gelman et al., 1996; Allen et al., 2007). In this latter approach body mass and cardiac output are assigned probability distributions, and masses and flows of organs and aggregated compartments are specified as proportions of body mass and cardiac output, respectively. There is an inconsistency in the literature as to the probability distributions assigned to these proportions. For example, Gelman et al. (1996) represented population variability using lognormal distributions whereas Allen et al. (2007) used normal distributions. Furthermore, when many compartments are discretely defined, logical constraints on mass balance and cardiac output can be violated. Gelman et al. (1996) proposed a re-parameterization for circumventing this problem. However, even when logical constraints on mass balance and cardiac output are not violated, this piecemeal approach defines a physiology that is much more diverse than actually occurs within the human population: a model that satisfies mass balance constraints does not necessarily equate to a physically realistic physiology. McNally et al. (2012) noted that due to the scarcity of data for calibration there was little difference between marginal prior distributions and posterior distributions for many parameters; the implication is therefore, that inference (on dose reconstruction) could have a strong dependence on (arbitrary) decisions of the modeler.

In the past two decades there has been substantial progress in the quantification of variability in human anatomical, physiological and biochemical parameters (Price et al., 2003; Willmann et al., 2007; Jamei et al., 2009). Freely available software, linked to population databases, such as Physiological Parameters for PBPK Modeling (P^3^M) (P^3^M™ Database, 2003), and PopGen^2^ (McNally et al., 2014) and commercially available software such as PK-Sim®^3^ and Simcyp^4^ can generate realistic anatomically correct human populations. Invariably these tools also generate a proportion of implausible physiologies although this is much smaller than for the approaches described previously. However, whilst software for generating a virtual human population is available, at present, a concise probabilistic description of human variability (i.e., not executed in a computer code) is not readily available. These software applications can generate records which can be read into software and therefore used in conventional forward dosimetry, however the algorithms are not in a mathematically convenient form that allow precise prior distributions to be easily specified. Population summaries from these applications can be used (McNally et al., 2014) and offer an improvement over the prior specifications found in the literature, however population summaries fail to capture key correlations and therefore allow unrealistic variability in physiology.

The present work aims to plug the gap between software algorithms and reverse dosimetry applications by developing a prior distribution for the physiology of the adult working population (individuals aged between 16 and 65) that provides narrower and more realistic bounds on the human physiology. We consider male and female UK and US populations. Our prior distributions vary by gender but also by ethnicity; we provide prior distributions for Caucasian, Asian and Black UK populations and Caucasian, Non-Black Hispanic, and Black US populations. Some comparisons against alternative prior specifications from the literature are made. Moreover, we report on how readily obtainable physical information such as gender, ethnicity, age and height can be used to substantially reduce uncertainty. Practical use of this prior specification in reverse dosimetry applications, and comparisons against previous results will be presented in a subsequent report.

## Materials and methods

### Probability model for human variability

#### Conceptual approach

The model described in the paper is based upon the PopGen software developed at the Health and Safety Laboratory (McNally et al., 2014). PopGen simulates the physiology of healthy human populations. A two-phased approach is encoded in the software:

- In the first phase the gender, ethnicity, age, height, body mass, and cardiac output of an individual are generated.
- For an individual of a given gender, ethnicity, age, height, and weight the organ masses and flows are then generated in the second phase.

The software links to four reference databases covering UK, US, and Western European populations, which govern the gender and ethnicity dependent relationship between age, height, and body mass in the population. For the current work male and female populations were generated for a healthy working age UK population (ages 16 to 65) using the Health Survey for England (HSE) reference database (Department of Health, 2010). Ten thousand individuals were simulated for male and female populations for Caucasian, Asian and Black populations—six distinct cases in all. Male and female populations for a healthy working age US population were based upon the NHANES III reference database. Ten thousand individuals were generated for male and female populations for Caucasian, Non-Black Hispanic and Black populations. The populations were constrained to a Body Mass Index (BMI) in the range 17.5–32.5; the adaptations that are required for a grossly obese sub-population are discussed later. Our approach was based upon a statistical analysis of the output from each of these sub-populations.

The PopGen algorithms for organ masses and flows were initially based upon allometric scaling (based upon height) of reference man (ICRP, 2003) and replicated the methodology described in Willmann et al. (2007) as implemented in the PK-Sim®commercial software. Some changes to the relationships for organ masses have recently been made in the PopGen software and additionally it accounts for age-related trends in the adult population (Beaudouin et al., 2010), which are particularly important for cardiac output, skeletal muscle mass, skeleton mass, and adipose mass. Technical details can be found in McNally et al. (2014).

The key considerations in constructing a statistical approximation to the PopGen model were that: the marginal probability distributions that represent organ masses and flows needed to be a close approximation to the PopGen output; significant correlations between the masses and flows in the PopGen output needed to be appropriately modeled in the approximation; the framework should have sufficient flexibility to incorporate contextual information that allows tighter bounds on human physiology to be built into the prior specification. Contextual information includes determinants such as gender, age, weight, ethnicity, and height. Although contextual information is not required for a PBPK model this information is central to our approach. In essence we construct a viable human “shell” (phase 1) and then specify the organs and tissues within (phase 2). Contextual information may be probabilistically modeled, or may be replaced with known values or ranges.

#### Phase 1 parameters

Gender and ethnicity are assumed to be known for any given individual. The age of each population member is initially specified. For a probabilistically modeled age a non-standard distribution based on census data could be encoded, however, this very high level of precision is unnecessary. A piecewise linear fit is used in PopGen to smooth over small variations and to remove the need to annually update the population distribution; however a uniform distribution between 16 and 65 is an adequate approximation to the working age population (Table 1). Specific information about a population can easily replace this prior distribution, an exact age for study participants may be used, or limits for each individual (e.g., individual is known to be between 46 and 50 years of age) may be used.

**Table 1:** **Marginal distributions for age, height, and log(body mass) and the correlation between the height and log(body mass)**.

**Population**	**Age**	**Height (cm)**				**Log(Body Mass (kg))**				**Correlation**
		$H\;\sim \;N(\alpha +a_{1}A+a_{2}A^{2},\sigma^{2})$				$\text{log}(BM)\;\sim \;N(\alpha +a_{1}A+a_{2}A^{2},\sigma^{2})$				
		**α**	**a_1_**	**a_2_**	**σ**	**α**	**a_1_**	**a_2_**	**σ**	**ρ**
**UK**										
Male, White	U(16, 65)	176.0	0.15	−0.003	6.9	4.06	0.0168	−0.00017	0.17	0.41
Male, Asian		174.4	−0.086	0	6.3	3.96	0.0186	−0.00021	0.18	0.42
Male, Black		178.8	−0.113	0	7.4	4.10	0.0167	−0.0002	0.19	0.45
Female, White		163.9	0.052	−0.0017	6.3	4.00	0.0099	−0.00009	0.20	0.29
Female, Asian		162.0	−0.126	0	6.0	3.94	0.009	−0.00007	0.20	0.29
Female, Black		161.9	0.228	−0.0049	6.3	3.93	0.0147	−0.00013	0.19	0.36
**US**										
Male, White	U(16, 65)	175.6	0.136	−0.002	6.6	3.96	0.0197	−0.00020	0.19	0.43
Male, Non-black hispanic		171.0	−0.03	0	6.4	3.86	0.0226	−0.00024	0.18	0.44
Male, Black		172.4	0.267	−0.0038	7.0	3.92	0.0233	−0.00026	0.22	0.28
Female, White		162.1	0.142	−0.0025	6.3	3.88	0.014	−0.00012	0.22	0.31
Female, Non-black hispanic		159.3	−0.066	0	6.0	3.79	0.020	−0.00021	0.21	0.29
Female, Black		162.3	0.108	−0.0019	6.4	3.87	0.020	−0.0002	0.25	0.46

**Figure 2:**
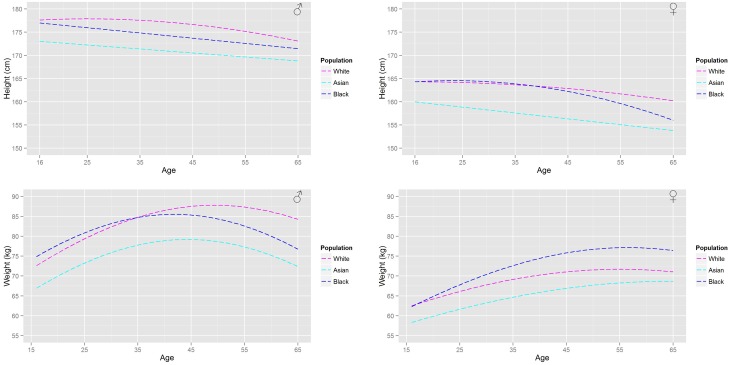
**A comparison of the age related trends in height and body mass for a UK population based upon the HSE survey**.

**Figure 3:**
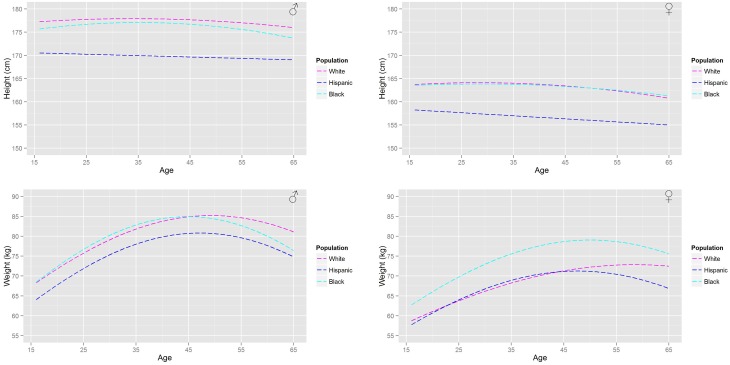
**A comparison of the age related trends in height and body mass for a US population based upon the NHANESIII survey**.

For a given ethnicity, gender, and age the height and weight are specified. An analysis of the reference databases indicated that heights are normally distributed with an age, gender, and ethnicity dependent mean. The relationship along with gender and ethnicity dependent coefficients are given in Table 1, where the upper case A denotes the age variable and the lower case characters denote coefficients. Similarly, the natural logs of body weight are normally distributed with age, gender, and ethnicity dependent means. The relationship and coefficients are also given in Table 1. A comparison of the age-related trends in height and body weight is made for a UK population in Figure 2 and for a US population in Figure 3. For heights the trend represents the arithmetic mean whereas for body weight the trend represents the geometric mean. After removing age related trends by regressing height and log body mass against age for each of the sub populations a large correlation between the residuals from these regression models residuals is apparent (Table 1); it is therefore necessary to simulate height and log body mass from a bivariate normal distribution in order to capture the relationship. A convenient way of specifying height and log body weight is to use a well-known formulation of the bivariate normal distribution

(1) $$X\;\sim \;N(\mu_{X},\sigma_{X}^{2})$$

(2) $$Y\sim N(\mu_{Y}+(\sigma_{Y}\sigma_{X}^{-1}\rho \left(X-\mu_{X}\right),\left(1-\rho^{2}\right)\sigma_{Y}^{2})$$

In Equations (1) and (2) μ_*X*_ and μ_*y*_ denote means, σ_*X*_ and σ_*y*_ denote standard deviations and ρ denotes the correlation. Parameter values are given in Table 1 for each sub population. The order of the simulations, i.e., unconditional height (Equation 1) and log body mass conditional on height (Equation 2) or vice-versa depends upon what contextual information may be available for an individual. Bounding information such as height or body mass known within an interval (for example a body mass between 65 and 70 kg) can easily be accommodated by specifying truncated normal distributions. The quantity σ^−1^(upper—lower) is computed for both height and log body mass, where upper and lower are as defaults taken as ± 3σ values and overwritten by known values where available: the variable for which this quantity is smallest is simulated unconditionally from Equation (1). The known heights and body weight of an individual remove the need for this probabilistic specification; the exact height and body mass would be used when defining organ and tissue masses and flows in phase 2 (described below). An example based upon bounding information is described in the results section.

The final phase 1 parameter is cardiac output. The trend in cardiac output from new-born to adulthood encoded into PopGen is based upon reference man (ICRP, 2003). An age-related decline in cardiac output is also encoded in the software. Additionally cardiac output scales with height. Equations describing cardiac output as a function of Age and Height are given in Table 2, with ethnicity dependent coefficients. In this instance different relationships were required for males and females owing to females reaching physical maturity sooner than males.

**Table 2:** **Probabilistic relationships between cardiac output and anthropometric (tier 1) parameters**.

**Population**	**Cardiac Output (***ml min***^**−1**^)**											
	**Male**						**Female**					
	CO ~ N(α + h_1_H + a_1_A + a_2_/(1+exp(-a_3_^*^(A-18))),σ^2^)						**CO ~ N(α + h_1_H + a_1_A + a_2_A^2^ + a_3_A^3^, σ^2^)**					
	**α**	**h_1_**	**a_1_**	**a_2_**	**a_3_**	**σ**	**α**	**h_1_**	**a_1_**	**a_2_**	**a_3_**	**σ**
**UK**												
White	2.755	0.025	−0.030	−0.707	−0.626	0.109	1.870	0.0246	0.0122	−0.000908	0.00000689	0.093
Asian	2.748	0.026	−0.031	−0.513	−0.716	0.108	1.703	0.0257	0.0173	−0.000916	0.00000642	0.093
Black	2.645	0.026	−0.029	−0.399	−0.934	0.110	1.893	0.0248	0.0090	−0.000869	0.00000689	0.093
**US**												
White	2.749	0.025	−0.031	−0.449	−0.820	0.110	1.854	0.0248	0.0127	−0.000956	0.00000739	0.092
Non−black hispanic	2.781	0.026	−0.031	−0.458	−0.828	0.109	1.792	0.0257	0.0166	−0.001015	0.00000765	0.092
Black	2.342	0.026	−0.031	0.403	0.917	0.111	1.903	0.0246	0.0088	−0.000839	0.00000625	0.093

#### Phase 2 parameters

In phase 2 organ and tissue masses and flows are specified based upon the phase 1 parameters. Regression models were fit to the data from each of the populations with the simulated organ flows as dependent variables and polynomials of the contextual variables [Age, Height, Body Mass, Body Mass Index (BMI; derived from height and body mass) and Cardiac Output] as explanatory variables. All flows were normally distributed and specified as fractions of cardiac output (Table 3). In contrast whilst the majority of organ masses were normally distributed, the masses of the lung, spleen, adipose, and skeletal muscle were log-normally distributed. Whilst the distributions were based on statistical analysis of data generated by PopGen, the PopGen algorithms themselves are informed by cadaver studies (Willmann et al., 2007). The mathematical forms, assumed distributions and dependencies are given in Table 3, with corresponding coefficients given in Tables 4A–C (UK populations) and Tables 5A–C (US populations). The variables A, H, BM, BMI, and CO denote Age, Height, Body Mass, BMI, and Cardiac Output whereas coefficients corresponding to these quantities are denoted using lower case letters.

**Table 3:** **Probabilistic relationships between anthropometric parameters simulated in stage 1 and organ masses and flows**.

**Organ**	**Mass (kg)**			**Flow (lmin**^**−1**^**)**		
	**Distribution**	**Dependencies**	**Form**	**Distribution**	**Dependencies**	**Form**
Lung	Log-normal	Height	α + *h*_1_*H*	Normal	CO	*c*_1_*CO*
Brain	Normal	Age	α + *a*_1_*A*	Normal	CO	*c*_1_*CO*
Heart	Normal	Height	α + *h*_1_*H*	Normal	CO	*c*_1_*CO*
Kidneys	Normal	Height	α + *h*_1_*H*	Normal	CO	*c*_1_*CO*
Liver	Normal	Height	α + *h*_1_*H*	Normal	CO	*c*_1_*CO*
Pancreas	Normal	Height	α + *h*_1_*H*	Normal	CO	*c*_1_*CO*
Spleen	Log-normal	Height	α + *h*_1_*H*	Normal	CO	*c*_1_*CO*
Stomach	Normal	Height	α + *h*_1_*H*	Normal	CO	*c*_1_*CO*
Small intestine	Normal	Height	α + *h*_1_*H*	Normal	CO	*c*_1_*CO*
Large intestine	Normal	Height	α + *h*_1_*H*	Normal	CO	*c*_1_*CO*
Sexual organs	Normal	None	α	Normal	CO	*c*_1_*CO*
Skin	Normal	Body mass	$\alpha +m_{1}BM+m_{2}BM^{2}$	Normal	CO	*c*_1_*CO*
Bone (M) Bone (F)	Normal	Age, Height	$\begin{array}{l} \alpha +h_{1}H+h_{2}H(1+\exp{\left(-a_{1}\left(A-18\right))\right)}^{-1}\\ \alpha +a_{1}A+a_{2}A^{2}+\;h_{1}H \end{array}$	Normal	CO	*c*_1_*CO*
Adipose	Log-normal	Age, Height, BMI	$\alpha +a_{1}A+a_{2}A^{2}+h_{1}H+b_{1}BMI$	Normal	CO	*c*_1_*CO*
Muscle	Log-normal	Age, Height, BMI	$\alpha +a_{1}A+a_{2}A^{2}+h_{1}H+b_{1}BMI+b_{2}BMI^{2}$	Normal	CO	*c*_1_*CO*

**Table 4A:** **Parameters for males and females in a UK Caucasian population**.

**Organ**	**Males**			**Females**		
	**Mass (kg)**		**Flow**	**Mass (kg)**		**Flow**
Lung	α = −0.503, *h*_1_ = 0.0044, σ = 0.324		*c*_1_ = 0.025, σ = 0.01	α = −0.565, *h*_1_ = 0.0037, σ = 0.325		*c*_1_ = 0.025, σ = 0.01
Brain	α = 1.488, *a*_1_ = −0.0023, σ = 0.070		*c*_1_ = 0.128, σ = 0.036	α = 1.374, *a*_1_ = −0.0021, σ = 0.065		*c*_1_ = 0.132, σ = 0.033
Heart	α = 0.094, *h*_1_ = 0.0019, σ = 0.108		*c*_1_ = 0.043, σ = 0.013	α = 0.116, *h*_1_ = 0.0014, σ = 0.089		*c*_1_ = 0.051, σ = 0.014
Kidneys	α = 0.128, *h*_1_ = 0.0019, σ = 0.116		*c*_1_ = 0.217, σ = 0.055	α = 0.137, *h*_1_ = 0.0018, σ = 0.107		*c*_1_ = 0.204, σ = 0.046
Liver	α = 0.689, *h*_1_ = 0.010, σ = 0.591		*c*_1_ = 0.069, σ = 0.021	α = 0.629, *h*_1_ = 0.0087, σ = 0.516		*c*_1_ = 0.071, σ = 0.019
Pancreas	α = 0.064, *h*_1_ = 0.0008, σ = 0.054		*c*_1_ = 0.011, σ = 0.003	α = 0.031, *h*_1_ = 0.0009, σ = 0.052		*c*_1_ = 0.010, σ = 0.003
Spleen	α = −2.272, *h*_1_ = 0.0047, σ = 0.368		*c*_1_ = 0.032, σ = 0.010	α = −2.309, *h*_1_ = 0.0049, σ = 0.364		*c*_1_ = 0.031, σ = 0.008
Stomach	α = 0.067, *h*_1_ = 0.0006, σ = 0.055		*c*_1_ = 0.011, σ = 0.003	α = 0.055, *h*_1_ = 0.00073, σ = 0.022		*c*_1_ = 0.010, σ = 0.003
Small intestine	α = 0.194, *h*_1_ = 0.003, σ = 0.092		*c*_1_ = 0.11 σ = 0.03	α = 0.205, *h*_1_ = 0.003, σ = 0.100		*c*_1_ = 0.122, σ = 0.031
Large intestine	α = 0.139, *h*_1_ = 0.002, σ = 0.086		*c*_1_ = 0.043, σ = 0.013	α = 0.109, *h*_1_ = 0.0020, σ = 0.058		*c*_1_ = 0.051, σ = 0.014
Sexual organs	α = 0.0462, σ = 0.0027		*c*_1_ = 0.0005, σ = 0.0002	α = 0.012, σ = 0.0007		*c*_1_ = 0.0002, σ = 0.00006
Skin	α = 1.053, *m_1_* = 0.0464 *m_2_* = −0.00021, σ = 0.163		*c*_1_ = 0.053, σ = 0.016	α = 0.615, *m_1_* = 0.045, *m_2_* = −0.00024, σ = 0.166		*c*_1_ = 0.051, σ = 0.0139
Bone	α = 3.187, *a*_1_ = 0.379, *h*_1_ = 0.044, *h_2_* = 0.0098, σ = 0.128		*c*_1_ = 0.053, σ = 0.016	α = 1.801, *a*_1_ = 0.0377, *a*_2_ = −0.00045, *h*_1_ = 0.046, σ = 0.103		*c*_1_ = 0.051, σ = 0.014
Adipose	α = −3.51, *a*_1_ = −0.0115, *a*_2_ = 0.00016, *h*_1_ = 0.023, *b*_1_ = 0.105, σ = 0.328	ρ = −0.90	*c*_1_ = 0.053, σ = 0.016	α = −3.298, *a*_1_ = −0.0033, *a*_2_ = 0.00006, *h*_1_ = 0.024, *b*_1_ = 0.10, σ = 0.270	ρ = −0.9	*c*_1_ = 0.092, σ = 0.024
Muscle	α = 1.595, *a*_1_ = 0.0076, *a*_2_ = −0.00011, *h*_1_ = 0.0071, *b*_1_ = 0.0181, σ = 0.228		*c*_1_ = 0.181, σ = 0.048	α = 1.84, *a*_1_ = 0.003, *a*_2_ = −0.000055, *h*_1_ = 0.006, *b*_1_ = 0.0072, σ = 0.256		*c*_1_ = 0.122, σ = 0.031

**Table 4B:** **Parameters for males and females in a UK Asian population**.

**Organ**	**Males**			**Females**		
	**Mass (kg)**		**Flow**	**Mass (kg)**		**Flow**
Lung	α = −0.401, *h*_1_ = 0.0035, σ = 0.323		*c*_1_ = 0.025, σ = 0.01	α = −0.725, *h*_1_ = 0.0044, σ = 0.318		*c*_1_ = 0.025, σ = 0.01
Brain	α = 1.490, *a*_1_ = −0.0022, σ = 0.070		*c*_1_ = 0.128, σ = 0.036	α = 1.368, *a*_1_ = −0.0020, σ = 0.065		*c*_1_ = 0.132, σ = 0.033
Heart	α = 0.131, *h*_1_ = 0.0016, σ = 0.078		*c*_1_ = 0.043, σ = 0.013	α = 0.091, *h*_1_ = 0.0015, σ = 0.083		*c*_1_ = 0.051, σ = 0.014
Kidneys	α = 0.105, *h*_1_ = 0.0019, σ = 0.108		*c*_1_ = 0.217, σ = 0.055	α = 0.167, *h*_1_ = 0.0015, σ = 0.100		*c*_1_ = 0.204*, σ* = 0.046
Liver	α = 0.679, *h*_1_ = 0.0093, σ = 0.548		*c*_1_ = 0.069, σ = 0.021	α = 0.659, *h*_1_ = 0.0080, σ = 0.480		*c*_1_ = 0.071, σ = 0.019
Pancreas	α = 0.050, *h*_1_ = 0.0008, σ = 0.050		*c*_1_ = 0.011, σ = 0.003	α = 0.0373, *h*_1_ = 0.00083, σ = 0.048		*c*_1_ = 0.010, σ = 0.003
Spleen	α = −2.300, *h*_1_ = 0.0046, σ = 0.363		*c*_1_ = 0.032, σ = 0.010	α = −2.263, *h*_1_ = 0.0044, σ = 0.370		*c*_1_ = 0.031, σ = 0.008
Stomach	α = 0.055, *h*_1_ = 0.0006, σ = 0.051		*c*_1_ = 0.011, σ = 0.003	α = 0.057, *h*_1_ = 0.00067, σ = 0.021		*c*_1_ = 0.010, σ = 0.003
Small intestine	α = 0.204, *h*_1_ = 0.003, σ = 0.087		*c*_1_ = 0.11, σ = 0.03	α = 0.247, *h*_1_ = 0.003, σ = 0.093		*c*_1_ = 0.122, σ = 0.031
Large intestine	α = 0.120, *h*_1_ = 0.0016, σ = 0.080		*c*_1_ = 0.043, σ = 0.013	α = 0.139, *h*_1_ = 0.0017, σ = 0.054		*c*_1_ = 0.051, σ = 0.014
Sexual organs	α = 0.043, σ = 0.0024		*c*_1_ = 0.0005, σ = 0.0002	α = 0.012, σ = 0.0008		*c*_1_ = 0.0002, σ = 0.00006
Skin	α = 0.943, *m_1_* = 0.050, *m_2_* = −0.00024, σ = 0.158		*c*_1_ = 0.053, σ = 0.016	α = 0.377, *m_1_* = 0.052, *m_2_* = −0.0003, σ = 0.165		*c*_1_ = 0.051, σ = 0.0139
Bone	α = 2.880, *a*_1_ = 0.357, *h*_1_ = 0.0429, *h_2_* = 0.0097, σ = 0.121		*c*_1_ = 0.053, σ = 0.016	α = 1.451, *a*_1_ = 0.0464, *a*_2_ = −0.00051, *h*_1_ = 0.044, σ = 0.096		*c*_1_ = 0.051, σ = 0.014
Adipose	α = −3.658, *a*_1_ = −0.0126, *a*_2_ = 0.00018, *h*_1_ = 0.0242, *b*_1_ = 0.106, σ = 0.327	ρ = −0.9	*c*_1_ = 0.053, σ = 0.016	α = −3.29, *a*_1_ = −0.0080, *a*_2_ = 0.00011, *h*_1_ = 0.0247, *b*_1_ = 0.103, σ = 0.268	ρ = −0.87	*c*_1_ = 0.092, σ = 0.024
Muscle	α = 1.517, *a*_1_ = 0.0081, *a*_2_ = −0.00012, *h*_1_ = 0.0075, *b*_1_ = 0.017, σ = 0.227		*c*_1_ = 0.181, σ = 0.048	α = 1.644, *a*_1_ = 0.0062, *a*_2_ = −0.000094, *h*_1_ = 0.0067, *b*_1_ = 0.0071, σ = 0.251		*c*_1_ = 0.122, σ = 0.031

**Table 4C:** **Parameters for males and females in a UK Black population**.

**Organ**	**Males**			**Females**		
	**Mass (kg)**		**Flow (lmin^−1^)**	**Mass (kg)**		**Flow (lmin^−1^)**
Lung	α = −0.299 *h*_1_ = 0.0035, σ = 0.320		*c*_1_ = 0.025, σ = 0.01	α = −0.852, *h*_1_ = 0.0050, σ = 0.321		*c*_1_ = 0.025, σ = 0.01
Brain	α = 1.482, *a*_1_ = −0.0021, σ = 0.070		*c*_1_ = 0.128, σ = 0.036	α = 1.374, *a*_1_ = −0.0021, σ = 0.064		*c*_1_ = 0.132, σ = 0.033
Heart	α = 0.169, *h*_1_ = 0.0017, σ = 0.089		*c*_1_ = 0.043, σ = 0.013	α = 0.071, *h*_1_ = 0.0016, σ = 0.082		*c*_1_ = 0.051, σ = 0.014
Kidneys	α = 0.119, *h*_1_ = 0.002, σ = 0.121		*c*_1_ = 0.217, σ = 0.055	α = 0.128, *h*_1_ = 0.0017, σ = 0.099		*c*_1_ = 0.204*, σ* = 0.046
Liver	α = 0.867, *h*_1_ = 0.0098, σ = 0.621		*c*_1_ = 0.069, σ = 0.021	α = 0.528, *h*_1_ = 0.0084, σ = 0.474		*c*_1_ = 0.071, σ = 0.019
Pancreas	α = 0.071, *h*_1_ = 0.0008, σ = 0.057		*c*_1_ = 0.011, σ = 0.003	α = 0.0485, *h*_1_ = 0.00072, σ = 0.048		*c*_1_ = 0.010, σ = 0.003
Spleen	α = −2.155, *h*_1_ = 0.0044, σ = 0.368		*c*_1_ = 0.032, σ = 0.010	α = −2.490, *h*_1_ = 0.0056, σ = 0.368		*c*_1_ = 0.031, σ = 0.008
Stomach	α = 0.039, *h*_1_ = 0.0008, σ = 0.058		*c*_1_ = 0.011, σ = 0.003	α = 0.041, *h*_1_ = 0.00073, σ = 0.021		*c*_1_ = 0.010, σ = 0.003
Small intestine	α = 0.229, *h*_1_ = 0.0033, σ = 0.097		*c*_1_ = 0.11, σ = 0.03	α = 0.186, *h*_1_ = 0.003, σ = 0.093		*c*_1_ = 0.122, σ = 0.031
Large intestine	α = 0.148, *h*_1_ = 0.0017, σ = 0.089		*c*_1_ = 0.043, σ = 0.013	α = 0.101, *h*_1_ = 0.0019, σ = 0.053		*c*_1_ = 0.051, σ = 0.014
Sexual organs	α = 0.048, σ = 0.0028		*c*_1_ = 0.0005, σ = 0.0002	α = 0.012, σ = 0.0007		*c*_1_ = 0.0002, σ = 0.00006
Skin	α = 1.051, *m_1_* = 0.046, *m_2_* = −0.0002, σ = 0.153		*c*_1_ = 0.053, σ = 0.016	α = 0.615, *m_1_* = 0.0452, *m_2_* = −0.00024, σ = 0.186		*c*_1_ = 0.051, σ = 0.014
Bone	α = 3.564, *a*_1_ = 0.440, *h*_1_ = 0.051, *h_2_* = 0.0072, σ = 0.146		*c*_1_ = 0.053, σ = 0.016	α = 1.964, *a*_1_ = 0.024, *a*_2_ = −0.0003, *h*_1_ = 0.043, σ = 0.097		*c*_1_ = 0.051, σ = 0.014
Adipose	α = −3.835, *a*_1_ = −0.0069, *a*_2_ = 0.0001, *h*_1_ = 0.0241, *b*_1_ = 0.103, σ = 0.338	ρ = −0.9	*c*_1_ = 0.053, σ = 0.016	α = −2.836, *a*_1_ = −0.0016, *a*_2_ = 0.000038, *h*_1_ = 0.026, *b*_1_ = 0.096, σ = 0.237	ρ = −0.87	*c*_1_ = 0.092, σ = 0.024
Muscle	α = 1.465, *a*_1_ = 0.0052, *a*_2_ = −0.00008, *h*_1_ = 0.0076, *b*_1_ = 0.026, σ = 0.219		*c*_1_ = 0.181, σ = 0.048	α = 1.88, *a*_1_ = 0.0039, *a*_2_ = −0.000071, *h*_1_ = 0.0059, *b*_1_ = 0.0035, σ = 0.256		*c*_1_ = 0.122, σ = 0.031

**Table 5A:** **Parameters for males and females in a US Caucasian population**.

**Organ**	**Males**			**Females**		
	**Mass (kg)**		**Flow**	**Mass (kg)**		**Flow**
Lung	α = −0.582, *h*_1_ = 0.0047, σ = 0.324		*c*_1_ = 0.025, σ = 0.01	α = −0.661, *h*_1_ = 0.0037, σ = 0.323		*c*_1_ = 0.025, σ = 0.01
Brain	α = 1.486, *a*_1_ = −0.0022, σ = 0.069		*c*_1_ = 0.128, σ = 0.036	α = 1.370, *a*_1_ = −0.0021, σ = 0.065		*c*_1_ = 0.132, σ = 0.033
Heart	α = 0.098, *h*_1_ = 0.0018, σ = 0.081		*c*_1_ = 0.043, σ = 0.013	α = 0.078, *h*_1_ = 0.0015, σ = 0.081		*c*_1_ = 0.051, σ = 0.014
Kidneys	α = 0.086, *h*_1_ = 0.0020, σ = 0.113		*c*_1_ = 0.217, σ = 0.055	α = 0.102, *h*_1_ = 0.0018, σ = 0.100		*c*_1_ = 0.204*, σ* = 0.046
Liver	α = 0.189, *h*_1_ = 0.0124, σ = 0.571		*c*_1_ = 0.069, σ = 0.021	α = 0.386, *h*_1_ = 0.0092, σ = 0.471		*c*_1_ = 0.071, σ = 0.019
Pancreas	α = 0.035, *h*_1_ = 0.0009, σ = 0.053		*c*_1_ = 0.011, σ = 0.003	α = 0.052, *h*_1_ = 0.0007, σ = 0.048		*c*_1_ = 0.010, σ = 0.003
Spleen	α = −2.166, *h*_1_ = 0.0040, σ = 0.364		*c*_1_ = 0.032, σ = 0.010	α = −2.361, *h*_1_ = 0.0047, σ = 0.368		*c*_1_ = 0.031, σ = 0.008
Stomach	α = 0.038, *h*_1_ = 0.00075, σ = 0.053		*c*_1_ = 0.011, σ = 0.003	α = 0.045, *h*_1_ = 0.0007, σ = 0.021		*c*_1_ = 0.010, σ = 0.003
Small intestine	α = 0.189, *h*_1_ = 0.0031, σ = 0.090		*c*_1_ = 0.11 σ = 0.03	α = 0.168, *h*_1_ = 0.0033, σ = 0.092		*c*_1_ = 0.122, σ = 0.031
Large intestine	α = 0.126, *h*_1_ = 0.0016, σ = 0.083		*c*_1_ = 0.043, σ = 0.013	α = 0.108, *h*_1_ = 0.0018, σ = 0.052		*c*_1_ = 0.051, σ = 0.014
Sexual organs	α = 0.045, σ = 0.0026		*c*_1_ = 0.0005, σ = 0.0002	α = 0.012, σ = 0.0007		*c*_1_ = 0.0002, σ = 0.00006
Skin	α = 1.068, *m_1_* = 0.0462 *m_2_* = −0.00021, σ = 0.163		*c*_1_ = 0.053, σ = 0.016	α = 0.656, *m_1_* = 0.044, *m_2_* = −0.00024, σ = 0.176		*c*_1_ = 0.051, σ = 0.0139
Bone	α = 3.044, *a*_1_ = 0.445, *h_2_* = 0.043, *h_2_* = 0.0093, σ = 0.127		*c*_1_ = 0.053, σ = 0.016	α = 1.708, *a*_1_ = 0.0293, *a*_2_ = −0.00036, *h*_1_ = 0.042, σ = 0.092		*c*_1_ = 0.051, σ = 0.014
Adipose	α = −3.536, *a*_1_ = −0.0094, *a*_2_ = 0.00014, *h*_1_ = 0.0232, *b*_1_ = 0.0105, σ = 0.325	ρ = −0.9	*c*_1_ = 0.053, σ = 0.016	α = −2.70, *a*_1_ = −0.0035, *a*_2_ = 0.00006, *h*_1_ = 0.022, *b*_1_ = 0.096, σ = 0.240	ρ = −0.86	*c*_1_ = 0.092, σ = 0.024
Muscle	α = 1.719, *a*_1_ = 0.0072, *a*_2_ = −0.00011, *h*_1_ = 0.0067, *b*_1_ = 0.0155, σ = 0.234		*c*_1_ = 0.181, σ = 0.048	α = 1.71, *a*_1_ = 0.0058, *a*_2_ = −0.000095, *h*_1_ = 0.0066, *b*_1_ = 0.0036, σ = 0.262		*c*_1_ = 0.122, σ = 0.031

**Table 5B:** **Parameters for males and females in a US Non-black Hispanic population**.

**Organ**	**Males**			**Females**		
	**Mass (kg)**		**Flow**	**Mass (kg)**		**Flow**
Lung	α = −0.628, *h*_1_ = 0.0047, σ = 0.322		*c*_1_ = 0.025, σ = 0.01	α = −0.750, *h*_1_ = 0.0044, σ = 0.321		*c*_1_ = 0.025, σ = 0.01
Brain	α = 1.488, *a*_1_ = −0.0022, σ = 0.069		*c*_1_ = 0.128, σ = 0.036	α = 1.369, *a*_1_ = −0.0020, σ = 0.064		*c*_1_ = 0.132, σ = 0.033
Heart	α = 0.094, *h*_1_ = 0.0018, σ = 0.076		*c*_1_ = 0.043, σ = 0.013	α = 0.097, *h*_1_ = 0.0014, σ = 0.081		*c*_1_ = 0.051, σ = 0.014
Kidneys	α = 0.059, *h*_1_ = 0.0021, σ = 0.105		*c*_1_ = 0.217, σ = 0.055	α = 0.112, *h*_1_ = 0.0018, σ = 0.097		*c*_1_ = 0.204*, σ* = 0.046
Liver	α = 0.726, *h*_1_ = 0.0089, σ = 0.546		*c*_1_ = 0.069, σ = 0.021	α = 0.502, *h*_1_ = 0.0088, σ = 0.471		*c*_1_ = 0.071, σ = 0.019
Pancreas	α = 0.034, *h*_1_ = 0.0009, σ = 0.050		*c*_1_ = 0.011, σ = 0.003	α = 0.054, *h*_1_ = 0.0007, σ = 0.048		*c*_1_ = 0.010, σ = 0.003
Spleen	α = −2.201, *h*_1_ = 0.0040, σ = 0.370		*c*_1_ = 0.032, σ = 0.010	α = −2.349, *h*_1_ = 0.0049, σ = 0.366		*c*_1_ = 0.031, σ = 0.008
Stomach	α = 0.039, *h*_1_ = 0.0007, σ = 0.050		*c*_1_ = 0.011, σ = 0.003	α = 0.047, *h*_1_ = 0.0007, σ = 0.021		*c*_1_ = 0.010, σ = 0.003
Small intestine	α = 0.197, *h*_1_ = 0.0030, σ = 0.090		*c*_1_ = 0.11, σ = 0.03	α = 0.153, *h*_1_ = 0.0035, σ = 0.092		*c*_1_ = 0.122, σ = 0.031
Large intestine	α = 0.064, *h*_1_ = 0.0019, σ = 0.078		*c*_1_ = 0.043, σ = 0.013	α = 0.117, *h*_1_ = 0.0018, σ = 0.053		*c*_1_ = 0.051, σ = 0.014
Sexual organs	α = 0.046, σ = 0.0027		*c*_1_ = 0.0005, σ = 0.0002	α = 0.012, σ = 0.0008		*c*_1_ = 0.0002, σ = 0.00006
Skin	α = 0.956, *m_1_* = 0.0491, *m_2_* = −0.00023, σ = 0.166		*c*_1_ = 0.053, σ = 0.016	α = 0.545, *m_1_* = 0.0477, *m_2_* = −0.00027, σ = 0.174		*c*_1_ = 0.051, σ = 0.0139
Bone	α = 2.883, *a*_1_ = 0.528, *h*_1_ = 0.0405, *h_2_* = 0.0097, σ = 0.117		*c*_1_ = 0.053, σ = 0.016	α = 1.640, *a*_1_ = 0.040, *a*_2_ = −0.00042, *h*_1_ = 0.043, σ = 0.098		*c*_1_ = 0.051, σ = 0.014
Adipose	α = −3.52, *a*_1_ = −0.0105, *a*_2_ = 0.00015, *h*_1_ = 0.0235, *b*_1_ = 0.105, σ = 0.331	ρ = −0.9	*c*_1_ = 0.053, σ = 0.016	α = −3.161, *a*_1_ = −0.0040, *a*_2_ = 0.00007, *h*_1_ = 0.0235, *b*_1_ = 0.100, σ = 0.258	ρ = −0.87	*c*_1_ = 0.092, σ = 0.024
Muscle	α = 1.476, *a*_1_ = 0.0077, *a*_2_ = −0.00011, *h*_1_ = 0.0078, *b*_1_ = 0.017, σ = 0.233		*c*_1_ = 0.181, σ = 0.048	α = 1.70, *a*_1_ = 0.0053, *a*_2_ = −0.000087, *h*_1_ = 0.0063, *b*_1_ = 0.0072, σ = 0.256		*c*_1_ = 0.122, σ = 0.031

**Table 5C:** **Parameters for males and females in a US Black population**.

**Organ**	**Males**			**Females**		
	**Mass (kg)**		**Flow (lmin^−1^)**	**Mass (kg)**		**Flow (lmin^−1^)**
Lung	α = −0.603, *h*_1_ = 0.0049, σ = 0.325		*c*_1_ = 0.025, σ = 0.01	α = −0.830, *h*_1_ = 0.0053, σ = 0.322		*c*_1_ = 0.025, σ = 0.01
Brain	α = 1.490, *a*_1_ = −0.0023, σ = 0.070		*c*_1_ = 0.128, σ = 0.036	α = 1.367, *a*_1_ = −0.0020, σ = 0.065		*c*_1_ = 0.132, σ = 0.033
Heart	α = 0.119, *h*_1_ = 0.0018, σ = 0.082		*c*_1_ = 0.043, σ = 0.013	α = 0.108, *h*_1_ = 0.0015, σ = 0.088		*c*_1_ = 0.051, σ = 0.014
Kidneys	α = 0.127, *h*_1_ = 0.0019, σ = 0.113		*c*_1_ = 0.217, σ = 0.055	α = 0.086, *h*_1_ = 0.0021, σ = 0.110		*c*_1_ = 0.204, σ = 0.046
Liver	α = 0.841, *h*_1_ = 0.0090, σ = 0.586		*c*_1_ = 0.069, σ = 0.021	α = 0.528, *h*_1_ = 0.0084, σ = 0.474		*c*_1_ = 0.071, σ = 0.019
Pancreas	α = 0.041, *h*_1_ = 0.0009, σ = 0.053		*c*_1_ = 0.011, σ = 0.003	α = 0.033, *h*_1_ = 0.0009, σ = 0.053		*c*_1_ = 0.010, σ = 0.003
Spleen	α = −2.191, *h*_1_ = 0.0042, σ = 0.369		*c*_1_ = 0.032, σ = 0.010	α = −2.402, *h*_1_ = 0.0055, σ = 0.367		*c*_1_ = 0.031, σ = 0.008
Stomach	α = 0.029, *h*_1_ = 0.0008, σ = 0.053		*c*_1_ = 0.011, σ = 0.003	α = 0.051, *h*_1_ = 0.00076, σ = 0.023		*c*_1_ = 0.010, σ = 0.003
Small intestine	α = 0.165, *h*_1_ = 0.0034, σ = 0.092		*c*_1_ = 0.11, σ = 0.03	α = 0.158, *h*_1_ = 0.0038, σ = 0.102		*c*_1_ = 0.122, σ = 0.031
Large intestine	α = 0.111, *h*_1_ = 0.0018, σ = 0.086		*c*_1_ = 0.043, σ = 0.013	α = 0.097, *h*_1_ = 0.0021, σ = 0.058		*c*_1_ = 0.051, σ = 0.014
Sexual organs	α = 0.048, σ = 0.0028		*c*_1_ = 0.0005, σ = 0.0002	α = 0.012, σ = 0.0007		*c*_1_ = 0.0002, σ = 0.00006
Skin	α = 1.112, *m*_1_ = 0.0447, *m_2_* = −0.0002, σ = 0.156		*c*_1_ = 0.053, σ = 0.016	α = 0.564, *m*_1_ = 0.0462, *m_2_* = −0.00024, σ = 0.182		*c*_1_ = 0.051, σ = 0.014
Bone	α = 3.22, *a*_1_ = 0.635, *h*_1_ = 0.0474, *h*_2_ = 0.0076, σ = 0.149		*c*_1_ = 0.053, σ = 0.016	α = 2.11, *a*_1_ = 0.031, *a*_2_ = −0.00043, *h*_1_ = 0.047, σ = 0.107		*c*_1_ = 0.051, σ = 0.014
Adipose	α = −3.919, *a*_1_ = −0.0049, *a*_2_ = 0.00009, *h*_1_ = 0.0241, *b*_1_ = 0.108, σ = 0.326	ρ = −0.9	*c*_1_ = 0.053, σ = 0.016	α = −2.836, *a*_1_ = −0.0016, *a*_2_ = 0.000038, *h*_1_ = 0.026, *b*_1_ = 0.096, σ = 0.237	ρ = −0.87	*c*_1_ = 0.092, σ = 0.024
Muscle	α = 1.812, *a*_1_ = 0.0048, *a*_2_ = −0.00008, *h*_1_ = 0.0065, *b*_1_ = 0.0163, σ = 0.226		*c*_1_ = 0.181, σ = 0.048	α = 1.90, *a*_1_ = 0.0050, *a*_2_ = −0.000085, *h*_1_ = 0.0056, *b*_1_ = 0.0064, σ = 0.256		*c*_1_ = 0.122, σ = 0.031

Residuals from each of the regression models were compared to assess for correlations that were not accounted for by relationships between the phase 1 parameters. The only significant and indeed very strong negative correlation was between the adipose and skeletal muscle; excess mass in an individual is more likely to arise due to either a high muscle mass or high adipose mass, rather than a more even allocation of both (Willmann et al., 2007). The natural logs of adipose and skeletal muscle masses are therefore simulated from a bivariate normal distribution. The correlation coefficients are provided for each population in Tables 4A–C, 5A–C.

#### Monte-carlo simulation

Simulation of a member of a population follows the hierarchical framework described in Sections Phase 1 Parameters and Phase 2 Parameters. The age of each member of the population is initially simulated. Height and body mass are then jointly simulated (allowing BMI to be calculated) and finally cardiac output is simulated based upon the coefficients in Tables 1, 2, respectively. Organ flows, specified as a proportion of cardiac output, are then simulated (Table 2) and with intra-individual variability accounted for by simulating from normal distributions. Intra-individual variability in organ flows is not well understood and the ranges are probably generous therefore a truncation at two standard deviations is proposed. Flow balance is achieved by re-scaling all flows such that the ratio of their sum to that of cardiac output is unity. Organ masses are simulated using the expressions in Table 3 with population specific parameters given in Tables 4A–C, 5A–C with intra-individual variability represented using normal and log-normal distributions. A truncation at the 5th and 95th percentiles is proposed for each organ so that masses are not unrealistically small or large.

In order to satisfy the mass balance constraint a re-scaling of organ masses is necessary. Due to the very large variability in adipose and muscle masses, particularly large values for these two tissue masses would require a large proportional reduction in all organ masses, a reduction which could take some organ masses beneath physically realistic values. The method proposed in this work is to treat all masses except adipose and muscle mass as being correct and to rescale just the adipose and muscle masses such that mass balance is achieved. After re-scaling the adipose and skeletal muscle masses accounted for approximately 50–80% of body mass.

A simpler physiology than that described in this paper is often adequate for many studies; rather than specifying all organs and tissues, a proportion are summed into aggregated rapidly and slowly perfused “compartments.” This is easily accommodated by summing organ masses and flows at the end of the simulation. Although the output is simplified all of the dependencies are captured in the resulting output. R code for simulating the populations described in this paper is provided in Supplementary Material.

#### Adaption for inverse problems

The class of inverse problems inspiring this research require the estimation of external dose from a measurement series. For reverse dosimetry the measurements are from biomarker data whereas for QIVIVE the measurements will result from an *in vitro, in silico*, or *in chemico* system. In order to account for variability in human physiology when estimating external dose, it is necessary to specify the problem within a Bayesian framework. Typically, inference about external dose and other parameters is made using a Markov Chain Monte Carlo (MCMC) algorithm. In practice a single component update is coded, with the algorithm stepping through and updating each parameter in turn. In order to retain mass balance, a “sink” compartment is specified; this is not specifically updated but is recalculated to retain mass and flow balance for every parameter update. A similar solution can be adopted in our case. We propose that the skeletal muscle (or a slowly perfused compartment) is modified such that mass and flow balance are achieved

The aggregation of organs and tissues into rapidly and slowly perfused compartments poses a greater complication for inverse problems, since a probability distribution for the aggregated compartment is required and a mixed sum of log-normal and normal distributions has no closed solution. An approximation is therefore required, and the form of this approximation will depend on the organs and tissues contained in the aggregated compartment: it is model and application specific. This is beyond the scope of current work.

### Comparisons

#### An adult male population

Sohn et al. (2004) described a model for inhalation exposure to trichloroethylene. A four compartment PBPK model with liver, adipose and slowly and rapidly perfused compartments was used. All masses and flows were specified directly using log-normal distributions. The components of the prior distribution relating to masses and flows, taken from Table 1 of Sohn et al. (2004) are provided in Table 6. A corresponding US Caucasian male population aged 16–65 was created using our probabilistic model and masses and flows corresponding to the four compartments were derived. The organ volumes (l) of Sohn et al. (2004) were converted to masses (kg) using a 1 to 1 relationship. In both cases ten thousand samples were drawn for the comparison.

**Table 6:** **Prior specification for organ volumes and flows of an adult male population as used in (Sohn et al., 2004)**.

**Compartment**	**Volume (l)**	**Flow (l/min)**
Fat	LN(12.87, 2.1)	LN(0.3, 2.3)
Slowly perfuse tissue	LN(42.3, 2.06)	LN(0.81, 2.22)
Rapidly perfuse tissue	LN(12.94, 2.18)	LN(3.98, 2.22)
Liver	LN(2.18, 2.08)	LN(1.12, 2.3)

*Distributions parameterized by the GM and GSD*.

#### A female population of child bearing age

Allen et al. (2007) described a PBPK model for oral exposure to methylmercury for women of childbearing age (which was taken to be 16–49). The model was detailed and included brain, fat, gut, intestine, kidney, liver, and rapidly and slowly perfused compartments (in addition to plasma and red blood cells). Body mass was specified as a log normal distribution, cardiac output as a normal distribution (scaled to BW^0.75^) and tissue masses and regional flows were defined as respective fractions. All fractions were modeled as truncated normal distributions, with upper and lower truncation points taken from Clewell et al. (1999). Some details of the prior specification from Table 1 of Allen et al. (2007) are provided in Table 7.

**Table 7:** **Prior specification for organ volumes and flows of an adult male population as used in Allen et al. (2007)**.

**Parameter**	**Distribution**	**Bounding Interval**
Body Weight (BW) (kg)	LN(log(67.77), log(1.603))	(30.81, 139.9)
Cardiac Output (L/hr)	N(20, 10)	(6.8, 33.2)
**TISSUE VOLUME (AS FRACTION OF BW)**		
Liver volume	N(0.026, 0.013)	(0.006, 0.046)
Kidney volume	N(0.004, 0.002)	(0.0004, 0.008)
Brain volume	N(0.02, 0.01)	(0.002, 0.038)
Intestine volume	N(0.014, 0.007)	(0.001, 0.027)
Gut volume	N(0.017, 0.0085)	(0.009, 0.025)
Richly perfused tissue volume	N(0.10, 0.05)	(0.01, 0.190)
Slowly perfused tissue volume	N(0.35, 0.175)	(0.18, 0.52)
Adipose volume	N(0.273, 0.14)	(0.076, 0.47)
Remainder (non-perfused)	N(0.122, 0.061)	(0.012,0.23)
**PLASMA FLOW (AS FRACTION OF CARDIAC OUTPUT)**		
Liver flow	N(0.046, 0.023)	(0.01, 0.090)
Kidney flow	N(0.175, 0.0875)	(0.018,0.333)
Brain flow	N(0.114, 0.057)	(0.011, 0.217)
Gut flow	N(0.181, 0.0905)	(0.002, 0.360)
Richly perfused tissue flow	N(0.183, 0.0915)	(0.018, 0.348)
Slowly perfused tissue flow	N(0.249, 0.1245)	(0.025, 0.473)
Adipose flow	N(0.052, 0.0256)	(0.0052, 0.099)

*Upper and lower bounds taken from Clewell et al. (1999)*.

Two populations were generated using our prior distribution for comparison with Allen et al. (2007). Priors for a 16 year old Hispanic woman and for a 49 year old Caucasian woman were sampled from. The PopGen organ volumes include the mass of the blood whereas the plasma and red blood cells were independently modeled in Allen et al. (2007). To enable a like-for-like comparison a blood mass of 4.1 kg was assumed and the weight fraction of the blood was subtracted from all our tissues masses based upon reference values (ICRP, 2003). Ten thousand samples were drawn using our model and the priors of Allen et al. (2007).

In both examples described above each virtual member of the population was compared against the PopGen output generated to build the statistical models to assess for a physically realistic physiology: PopGen itself is informed by cadaver studies (Willmann et al., 2007). Individuals with any organ mass (or amalgamated compartment) outside the absolute bounds of the PopGen output were classed as physically unrealistic. Virtual individuals with a cardiac output outside of the PopGen range were also classified as physically unrealistic.

#### Bounding information

Earlier we described how exact information removes the need for a probabilistic representation for age, height, and weight. In this comparison we demonstrate how bounding information can provide a similar quality of contextual information and result in a substantial increase in the precision of the prior specification.

Prior distributions were generated for a UK Caucasian male. Additional contextual information was that this individual was aged between 50 and 55 years, between 1.71 and 1.75 m in height, and between 75 and 80 kg in body weight. Bounding information of this type would typically be obtained from tick box survey responses. In the first instance the contextual information was not utilized, however the contextual information about this individual was sequentially fed into the prior specification. Information on age, height, and mass were each used in isolation before all three pieces of information were utilized. Prior distributions for these five distinct cases were generated and compared.

## Results

### Populations

Figure 2 shows the (arithmetic) mean heights and (geometric) mean weights for a working age (16–65 years) male and female population from the HSE database. Figure 3 shows a similar plot for a US population from the NHANESIII database. In both figures the distinct trends for the main ethnicities within the respective UK and US populations can be distinguished.

These population databases show that the heights of US black and Caucasian populations have plateaued whereas the US Hispanic population continues to increase in height with each successive generation. In comparison only a plateau in heights can be seen within the Caucasian population in the UK with heights in the Asian and Black population still increasing. All populations (Figures 2, 3) show that individuals at the upper end of the age range have smaller heights. In comparison all populations show a substantial increase in body weight even after physical maturity is reached; in some populations body weights are lower at the upper end of the age range. The relationship between age and size (height and body mass) is clearly both gender and ethnicity dependent.

The differing relationships (Tables 3, 4A–C, 5C) that relate organ masses to age, height and body weight for each of the studied populations reflect the gender and ethnicity differences in the relationships between age and size. The largest differences between populations are in the relationships between age and the adipose and skeletal muscle masses. Large increases in the adipose mass after maturity are seen in all populations. Trends in the adipose mass closely follow the trends in body weights (Figures 2, 3).

**Figure 4:**
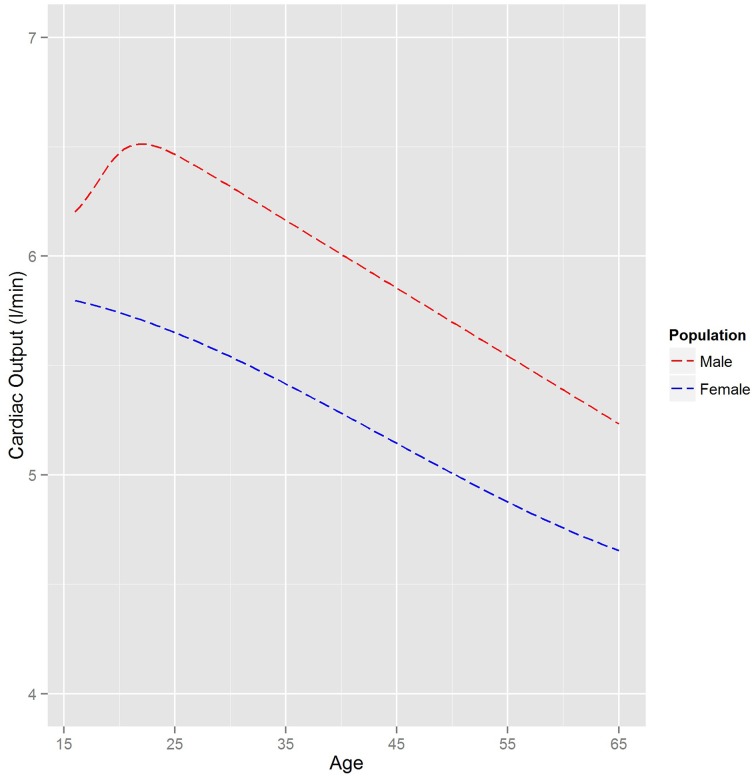
**The relationship between mean cardiac output and age for a UK Caucasian population**. The trends are based on heights of 180 and 160 cm for males and female, respectively.

Figure 4 shows a comparison of the relationships between age and (arithmetic) mean cardiac output for a UK Caucasian male and female population. Very prominent age dependencies can be seen with a difference in the trend for males and females between the ages of 16 and 25. The gender trends for males and females shown in Figure 4 are similar for other ethnicities.

### Comparisons

#### An adult male population

**Figure 5:**
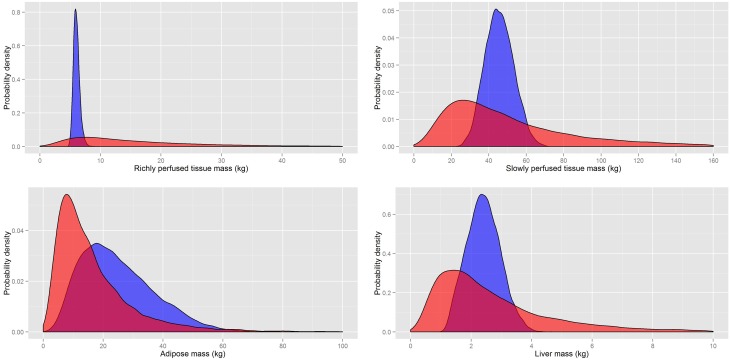
**A comparison of the prior distributions for the masses of rapidly and slowly perfused aggregated compartments and the adipose and liver masses from Sohn et al. (2004) (red) and based on PopGen (blue)**.

**Figure 6:**
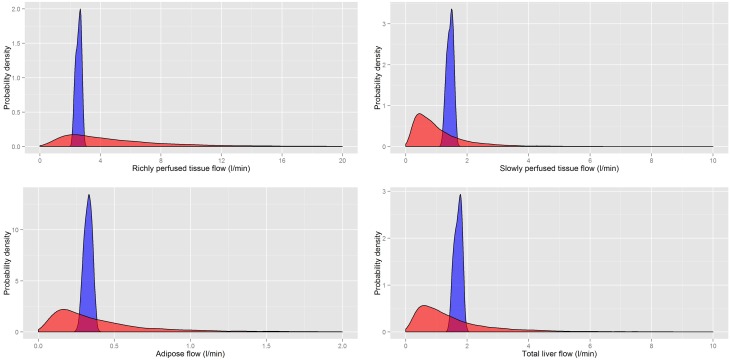
**A comparison of the prior distributions for the regional flows of rapidly and slowly perfused aggregated compartments, the adipose and the total liver flow (incorporating all flows discharging into the portal vein) from Sohn et al. (2004) (red) and based on PopGen (blue)**.

A comparison of the masses and flows from our probability distributions and those of Sohn et al. (2004) (Table 6) are made in Figures 5, 6, respectively. A comparison of the rapidly perfused, slowly perfused, adipose and liver tissue masses is made in Figure 5. A similar comparison of regional flows is made in Figure 6; note the total liver flow incorporates the regional flows from all tissues draining into the portal vein.

It is clear that for both masses and flows our distributions have much tighter limits with some differences in central tendency. While the prior distributions of Sohn et al. (2004) were sufficiently wide that they captured all reasonable human variability, a large proportion of all simulations resulted in masses and flows for individual compartments that were unrealistically large or small. Our analysis indicated that less than 1% of all generated physiologies using the prior distributions specified in Sohn et al. (2004) were consistent with human physiology.

Sohn et al. (2004) drew samples from their specified prior distributions and ran these parameter sets through the PBPK model. They noted huge variability (Figure 2 of Sohn et al., 2004) in their temporal forecasts of TCE concentrations in venous blood. Whilst it should be noted that partition coefficients, metabolic parameters, and three parameters describing exposure were also varied in their model, it is likely that excessive non-physical variability in human physiology made an important contribution to this very large variability in model forecasts.

#### A female population of child bearing age

A comparison of summary statistics (median and a 95% interval) for the organs and tissue masses (kg) and regional flows (L min^−1^) for a 16 year old Hispanic woman and a 49 year old Caucasian woman is made in Table 8. Summary statistics based upon the priors of Allen et al. (2007) are also provided.

**Table 8:** **Summary statistics (median values and 95% interval) for the physiology of a 16 year old Hispanic woman and a 49 year Caucasian women and corresponding summary statistics from the prior distributions of Allen et al. (2007)**.

**Parameter**	**Hispanic, aged 16**	**Caucasian, aged 49**	**Allen et al. (2007)**
Body weight (kg)	57.6 (41.9, 80.2)	68.2 (46.9, 89.5)	67.4 (34.0, 129.4)
Liver mass	1.49 (0.70, 2.42)	1.49 (0.67, 2.40)	1.67 (0.46, 4.31)
Kidney mass	0.32 (0.15, 0.50)	0.32 (0.15, 0.51)	0.26 (0.05, 0.70)
Brain mass	1.29 (1.18, 1.41)	1.21 (1.11, 1.35)	1.26 (0.27, 3.40)
Intestine mass	0.87 (0.68, 1.09)	0.87 (0.68, 1.09)	0.89 (0.16, 2.37)
Gut mass	0.12 (0.08, 0.16)	0.12 (0.08, 0.16)	1.12 (0.45, 2.57)
Rapidly perfused^a^ tissue mass	1.09 (0.61, 1.93)	1.09 (0.61, 1.93)	6.43 (1.23, 16.97)
Slowly perfused^b^ tissue mass	21.45 (12.91, 32.26)	21.0 (11.91, 32.40)	22.69 (9.18, 53.16)
Adipose mass	18.2 (6.34, 43.20)	29.4 (10.60, 54.50)	17.8 (4.30, 52.64)
Remainder (non-perfused)	8.70 (8.15, 9.3)	8.85 (8.31, 9.40)	7.8 (1.60, 21.0)
Cardiac Output (l/min)	5.90 (5.55, 6.25)	5.10 (4.75, 5.45)	7.6 (2.73, 16.3)
Liver flow	0.41 (0.37, 0.45)	0.35 (0.31, 0.39)	0.32 (0.076, 0.96)
Kidney flow	1.17 (1.08, 1.27)	1.02 (0.92, 1.11)	1.23 (0.22, 1.23)
Brain flow	0.76 (0.69, 0.82)	0.66 (0.59, 0.73)	0.80 (0.15, 2.34)
Gut flow^c^	1.06 (0.97, 1.14)	0.91 (0.83, 0.99)	1.28 (0.19, 3.84)
Rapidly perfused^a^ tissue flow	0.67 (0.62, 0.72)	0.58 (0.53, 0.64)	1.28 (0.24, 3.89)
Slowly perfused^b^ tissue flow	1.00 (0.91, 1.07)	0.86 (0.79, 0.94)	1.77 (0.31, 5.24)
Adipose flow	0.53 (0.48, 0.58)	0.46 (0.41, 0.51)	0.38 (0.05, 1.88)

a *Rapidly perfused compartment assumed to comprise of the heart, lung, spleen, pancreas and sexual organs*.

b *Slowly perfused compartment assumed to comprise of skin and skeletal muscle*.

c *Comprising of gut and intestine flows*.

These specific populations were chosen as they represent the bounding physiologies, a young woman of small stature and an older woman with larger stature, of the child bearing population defined in Allen et al. (2007). The summary statistics in Table 8 are based upon women with a BMI of between 17.5 and 32.5. Approximately 5 and 20% of the Hispanic and Caucasian populations respectively have a BMI in excess of 32.5 therefore we note these summaries are not representative of the wider populations, however the link between obesity and infertility has been well studied (Norman et al., 2004). The truncated populations considered here are reasonable surrogates for a child bearing sub-population.

There are some key physiological differences between the two simulated populations, which are relevant for the PBPK model for methylmercury. Cardiac output is greater for the 16 years old Hispanic woman since this peaks during female adolescence and subsequently declines (ICRP, 2003). As a consequence the regional blood flows, in particular those to the brain, kidneys and liver are lower in the 49 year old Caucasian. In terms of masses there is a large difference (approximately 10 kg) in the body weights of the two studied populations, which in part reflects the larger stature of the Caucasian population, but also accounts for the substantial gain in adipose tissue throughout adulthood (Table 8). The age related decline in brain mass can also be seen in summary statistics. There is of course a continuum between these two bounding cases but the example demonstrates how similar exposures might result in a different response in the different sub-populations. Note that in a simple comparison of the marginal distributions of individual organs the dependencies between organs (through age, height, and body weight) are not obvious.

Although truncated, the masses simulated from Allen et al. (2007) have greater variability than is seen in the general population; we would argue this range of masses represents unrealistically large variation in a child bearing population. The central values for organ masses were generally very close to ours, however for all organ masses, except the adipose mass, the simulated ranges from Allen et al. (2007) were unrealistically large, with 5th and 95th percentiles (Table 8) for each organ mass outside a physically realistic range. The variation in both cardiac output and in regional flows is well outside a physically realistic range.

We note there were two inconsistencies in our results which indicate potential errors in the specification of Allen et al. (2007). Whilst both gut and intestine masses are specified in Table 8, the summary statistics suggest the intestine mass is also accounted for within the gut. Additionally both the mass and regional flow to the rapidly perfused tissue appears to contain contributions from discretely defined organs and tissues. Even without these irregularities our work suggests that less than 1% of the physiologies generated using the priors of Allen et al. (2007) are within a physically realistic range.

#### Bounding information

**Figure 7:**
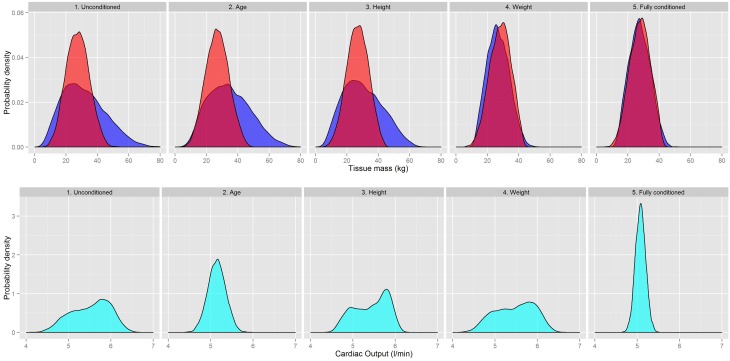
**A comparison of the Skeletal muscle (red) and Adipose masses (dark blue) and Cardiac Output (light blue) as different contextual information on age, body mass, and height is introduced into the prior specification**.

There were modest although statistically significant changes in all the organ masses resulting from the contextual information being input into the prior specification. However, sensitivity analysis (McNally et al., 2014) has demonstrated that even relatively modest changes to physiology can have an important impact on the pharmacokinetics of chemicals. The results are shown in Figure 7 for the skeletal muscle and adipose masses; these are the two largest tissue masses and also those with the greatest change as a result of the contextual information. Results for cardiac output are also shown.

Figure 7 shows there were changes in both the peak and dispersion of adipose mass as age and height information were utilized. The weight information had the greatest impact as this provided an upper bound on the adipose mass. Changes to the skeletal muscle were smaller than those for the adipose mass but the central estimate and dispersion of the distribution changed as the contextual information was input. The fully conditioned distributions were much narrower than the unconditioned distributions.

The marginal distribution for the cardiac output was particularly sensitive to age and height information. A much tighter distribution was obtained after incorporating all three pieces of contextual information. The changes to regional blood flows followed the same trends of cardiac output.

## Discussion

We noted in the introduction that there in an inconsistency in how human variability is specified in reverse dosimetry applications in the literature, in both the general approach adopted, and in the probability distributions that are specified. These varied approaches all define a potentially large proportion of parameter space that is inconsistent with human physiology. Our calculations in Sections Populations and Comparisons indicated that less than 1% of the physiologies generated using the prior distributions of Sohn et al. (2004) (Table 6) and Allen et al. (2007) (Table 7) were consistent with human physiology. These examples were chosen since the respective PBPK models encoded different levels of biological detail, and differed in the mathematical structure of the prior distributions. It is important to note that the examples were not “cherry picked”: we believe similar conclusions would be drawn if alternative prior distributions defined in the literature were subjected to similar scrutiny.

One important factor that influences the varying approaches described above is that the structure of a PBPK model is application specific. The number of organs and tissues explicitly defined depend upon the route of exposure, dose pattern, physico-chemical properties of the substance, and any additional cellular level behavior to be described within target organs. In contrast the methodology described in the current paper is not based upon a specific application and proposes distributions for a large number of tissues: our work can be simplified and adapted as necessary.

The use of probability to represent variability in the human population certainly cannot be described as novel, however as far as the authors are aware the link between population databases and the literature on physiological changes associated with aging has not been previously made. Furthermore, whilst differences between males and females are routinely accounted for in other studies, we are unaware of previous work that accounts for either age or ethnicity dependent differences in physiology. The ethnicity dependent distributions that have been developed in this work are a further complication, however the trends highlighted in Figures 1, 2 demonstrate that these are necessary for accurate population based inferences. However, despite the additional tier to the model which specifies age height and body weight, the organs and tissues themselves are all modeled using truncated normal and log-normal distributions, albeit with some dependencies.

The novel aspect of this research is in the use of contextual information. We demonstrated how even a single piece of contextual information on the age, height, or weight of a participant might be included to provide tighter bounds on the physiology of any individual. This feature could be utilized in population based studies where contextual information about participants might be available, either estimated by a researcher or from tick box responses to a survey. Additionally the probabilistic model can be tuned so that physiologies for a particular at-risk sub population (for example employees in a high hazard industry) can be generated.

Whilst the hierarchical approach has been justified there is room for further refinement in the models for organs and tissues. A proportion of parameter space defined by our prior distribution undoubtedly corresponds to physiologies that are inconsistent with a healthy human population. More precise relationships between organ masses and age, height, body weight and BMI and organ masses can be easily accommodated. The greatest scope for increased precision is offered by a more appropriate measure of “size.” Height is not a particularly useful measurement as very large differences in physiology are possible for individuals of a similar height. The fat free mass of the torso probably has a closer relationship with the internal organ masses. In principle, relationships between the length of the torso and internal organ masses could be established. This would require an additional tier in the model, linking height to torso length, for which anthropometric data is available. However, we are unaware of datasets relating torso length to organ masses; this approach would therefore need to be largely informed by autopsy data.

There is also the potential to further refine the modeling of regional blood flows. Mean values for the regional blood flows are based upon reference values (ICRP, 2003) and draw on the work of Williams and Leggett (Williams and Leggett, 1989; Leggett and Williams, 1991, 1995). Only modest variation in these values was coded. There have been significant advances in measurement technology since these reviews were conducted and recent studies of regional blood flow are available (Casey et al., 2008; Durduran and Yodh, 2014). An up-to-date systematic review of the recent literature in this area would be a valuable addition to the literature and could inform on more appropriate central estimates and variability for regional blood flow. A more precise physiology might be achieved by introducing a dependency between organ masses and flows. At present these are fully independent however, in principle a modest correlation could be easily supported, for example by truncating the regional flow to be strictly greater than average if the organ mass is also above average. This refinement is only likely to improve models that require a detailed physiology as the dependencies would be summed over if organs are aggregated in deriving rapidly and slowly perfused compartments.

A further area for refinement is in the modeling of obese populations. An upper BMI limit of 32.5 was used in developing the relationships in the current work although a modest extrapolation above this value this probably reasonable. Whilst the threshold BMI was somewhat arbitrary it is clear that a modification to our probabilistic model would be required for severely obese individuals. Whilst these modifications have not been thoroughly researched the anticipated changes that would be required can be outlined. The skeletal muscle mass would be generated independently from the adipose tissue with a different form for the mean and a truncation to the upper tail of the distribution (since excess mass will not result from the skeletal muscle); a different form for the skin mass would be required (probably a log-normal distribution) and a correlation with adipose mass would need to be coded; an increase in cardiac output, and increased regional flows to the skin and adipose tissues would be required.

Finally we note that inter-individual variability in bio-chemical parameters has not been considered in current research. Age related variation in metabolism is accounted for by PopGen and can be easily included in prior specifications (McNally et al., 2014). We are unaware of work that links human anthropometry to perfusion rates between the blood and organs.

The application to forward and reverse dosimetry problems (and other inverse problems such as QIVIVE) is a focus of current research. Whilst our work has not yet been demonstrated in conjunction with a PBPK model for either forward and reverse dosimetry applications (although this work is ongoing), the comparisons made against the prior distributions of Sohn et al. (2004) and Allen et al. (2007) are promising. A large range or distribution of external doses may be consistent with observed biological monitoring data, even when good quality information is available from a controlled laboratory-based study. This is the case since measurements only indirectly inform about the underlying parameters of the PBPK model through a comparison of model predictions and measurements at a number of time points. Similar forecasts in the time dependent model output can result from a wide range of exposures (in both dose, and duration) in combination with changes to the sensitive physiological parameters in the model. McNally et al. (2012) noted that with regard to physiological parameters, typically there is little difference in the marginal posterior distributions compared with the priors, although the large correlations between some parameters in the posterior do define a narrower range of physiologies that are consistent with the data compared with the prior. A subset of parameter space that is consistent with measurements will inevitably correspond to an unrealistic human physiology. The PBPK model, whilst physically based is ultimately just a model and will return forecasts even when the physiology defined by the input parameters is grossly inconsistent with a healthy human. For ill-posed inverse problems of this nature, the cautious Bayesian approach, of specifying wide priors which are subsequently refined using measurements, is flawed due to weak data. Prior specification therefore needs to discriminate between realistic and unrealistic physiologies. We do recognize that some physico-chemical parameters could be highly uncertain and will require conservative limits. Potentially these limits could be reassessed in a *post-hoc* analysis.

It was noted in Section Adaption for Inverse Problems that the aggregation of tissues into rapidly and slowly perfused compartments is not straight forward for inverse problems since a sum of normal and log-normal distributions does not have a closed-form probability distribution: this issue therefore relates to masses but not flows. In the discussion we focus on the rapidly perfused compartment although the slowly perfused tissues are modeled with similar reasoning. Sensitivity analysis might indicate that perfusion rates for rapidly perfused tissues are sufficiently similar to warrant an aggregated rapidly perfused compartment, however simulations will typically be very sensitive to the mass of the rapidly perfused compartment. Other authors assume a standard probability distribution for the mass of the rapidly perfused compartment, either directly or as a proportion of body weight. Results could be quite sensitive to this approximation. One solution could be to retain the (log-normally distributed) lung and spleen and aggregate the remaining normally distributed tissues. Assuming a common perfusion rate for all rapidly perfused tissues would introduce an additional four parameters into the PBPK model for each individual. A less precise yet computationally cheaper approach would be to approximate the rapidly perfused compartment by a suitable probability distribution. This conceptual problem will be addressed in greater depth in forthcoming work.

In conclusion we have developed a prior distribution for human physiology based on information from population databases and cadaver studies. The contextual information on age, height, and body mass that can be fed into the prior is unique and comparisons against published studies indicate our prior distribution defines much tighter bounds on human physiology. Until the methodology has been applied to a range of PBPK models of differing complexity it is unclear to what extent the prior will influence the precision of reverse dosimetry—this is a focus of current work.

## Funding

This publication and the work it describes were funded by the Health and Safety Executive (HSE). It's contents, including any opinions and/or conclusions expressed, are those of the authors alone and do not necessarily reflect HSE policy.

### Conflict of interest statement

The authors declare that the research was conducted in the absence of any commercial or financial relationships that could be construed as a potential conflict of interest.

## Footnotes

^1^Models that describe sub-cellular activity also need extensive checking when ranges for the parameters are specified in order to ensure that the model output does not describe odd and non-physical behavior for a subset of parameter space. These checks are similar in spirit, but potentially far more complex than mass and flow balance checks.

^2^http://xnet.hsl.gov.uk/popgen (6/8/2014).

^3^http://www.systems-biology.com/products/pk-sim.html (6/8/2014).

^4^http://www.simcyp.com/ (6/8/2014).
